# Simultaneous Bilateral Femoral Neck Stress Fracture in a Young Stone Mason

**DOI:** 10.1155/2015/306246

**Published:** 2015-05-26

**Authors:** Nikhil A. Khadabadi, Kiran S. Patil

**Affiliations:** Department of Orthopaedics, Jawaharlal Nehru Medical College, KLE University, Nehru Nagar, Belgaum, Karnataka 590010, India

## Abstract

Unilateral stress fractures of the femoral neck are very uncommon and bilateral involvement is even rarer. They commonly occur in athletes, military recruits, older persons, or individuals with underlying metabolic disorders and very seldom in normal individuals. We present a rare case of simultaneous bilateral fracture neck of femur in a 25-year-old man who came with complaints of pain in bilateral groin for 1 month. There was no history of trauma or history suggestive of excessive activity prior to the onset of pain, but there was history of lifting heavy weights daily. On evaluation with MRI scan bilateral fracture of the femur neck was diagnosed and patient was operated on bilaterally with internal fixation done using dynamic hip screw. Patient then regained his routine activity over a period of 6 months and on follow-up at 1 year no avascular necrosis changes were seen in the femur head. We presented this case because of its unusual presentation and the diagnostic challenge it poses.

## 1. Introduction

Stress fractures of the femur neck are very uncommon and bilateral fractures are very rarely seen. These fractures commonly occur in athletes, military recruits, older persons, or individuals with underlying metabolic disorders and very seldom they are seen in normal individuals. They usually present with chronic groin pain and can be missed if thorough radiographic evaluation is not carried out and thus high index of suspicion is needed to diagnose these fractures. The differential diagnosis for groin pain is wide and includes femoral neck stress fracture, proximal femoral stress fracture, stress fracture of the pubis, adductor tendonitis, avulsion fractures and urological causes, and hernia. A physician must first rule out those causes which are potentially dangerous. To rule out femoral neck stress fracture a plain X-ray is usually ordered. A plain X-ray has the ability to diagnose a macrostress fracture but not a microstress fracture. In the case presented even though a macrostress fracture was present it was not seen on the plain X-ray. We present a rare case of bilateral femoral neck stress fracture presenting as bilateral groin pain in a young stone mason.

## 2. Case Report

A 25-year-old male came with complaints of pain in both groins for 1 month. Pain was more in the left groin compared to the right side. He is mason by occupation and his work involves lifting heavy objects such as stones but does not involve walking long distances. The weight of the stone was 10–15 kg and he had to lift the stones at least 3 to 4 hours a day amounting to a total of 100 to 150 stones. The pain was dull aching in nature, continuous, and nonradiating. Pain was aggravated on standing and walking. Patient was able to do all his routine activities but with pain. He was able to squat, sit, and walk. There was no history of any other comorbidities and past history and social history were not significant.

On clinical examination patient had an antalgic gait and there was no limb length discrepancy. There was tenderness present over the anterior joint line of both hip joints bilaterally. Extremes of rotation were painful with internal rotation being more severe. Power in both lower limbs was normal and neurovascular examination was normal.

**Figure 1:**
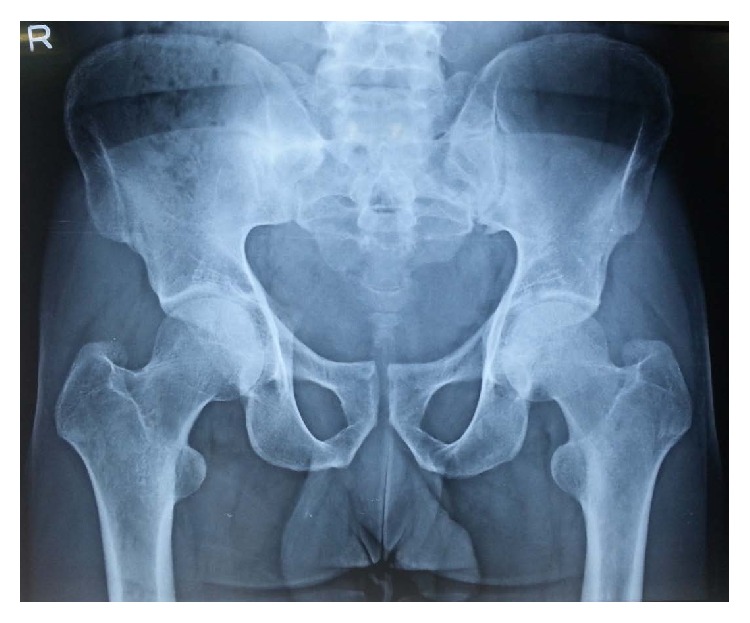
Plain radiograph of bilateral hip joint with pelvis demonstrating a faint fracture line in the left femur neck and no fracture line in the right femur neck.

**Figure 2:**
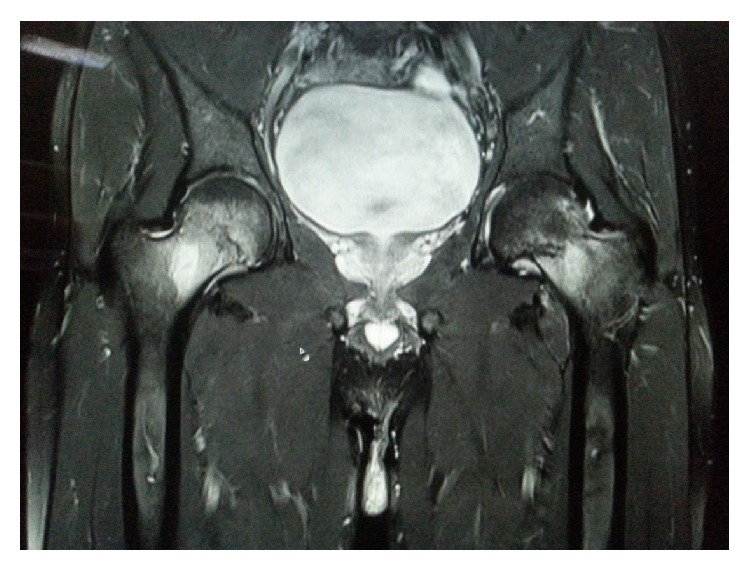
A T 2 weighted coronal magnetic resonance imaging (MRI) scan of pelvis with both hips showing undisplaced complete fracture on the left side and incomplete fracture on the right inferior neck.

**Figure 3:**
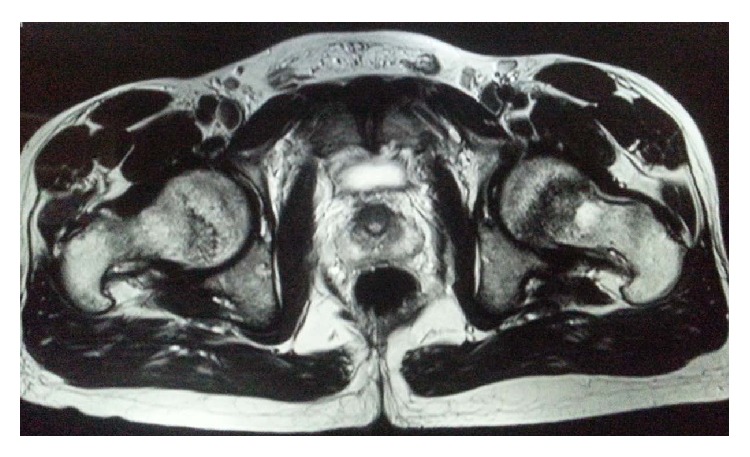
A T 2 weighted coronal magnetic resonance imaging (MRI) scan of pelvis with both hips showing undisplaced fracture of bilateral femur neck.

**Figure 4:**
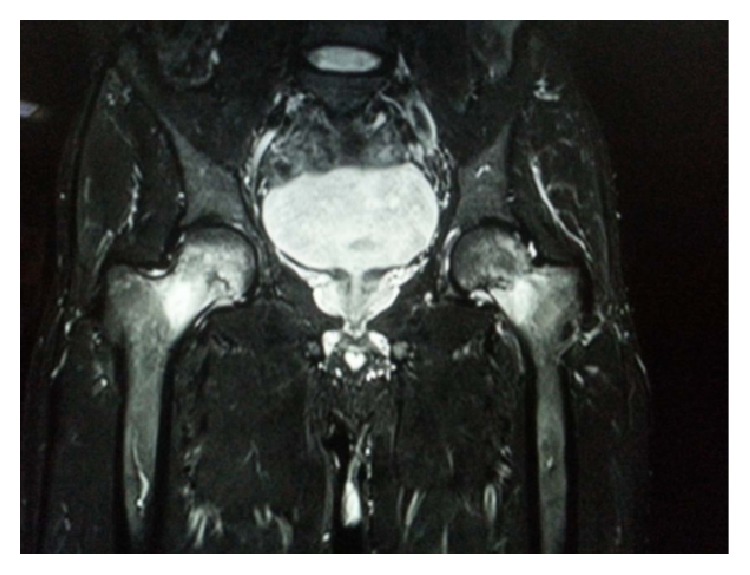
A T 2 weighted coronal magnetic resonance imaging (MRI) scan of pelvis with both hips showing oedema adjacent to the fracture line in the neck.

The patient was evaluated by X-ray of both hip joints which showed a possible fracture line in the femoral neck on the left and no abnormality on the right (Figure 1). On further investigation with MRI scan, a complete fracture line in the femur neck on the left side and an incomplete fracture line on the right side were seen (Figures 2, 3, and 4). Patient was admitted, immobilised, and evaluated for the stress fracture with blood tests and DEXA scan. The tests revealed a normal blood picture and no abnormality was seen on tests done to rule out metabolic disorders. DEXA scan of lumbar spine also showed a normal T score.

**Figure 5:**
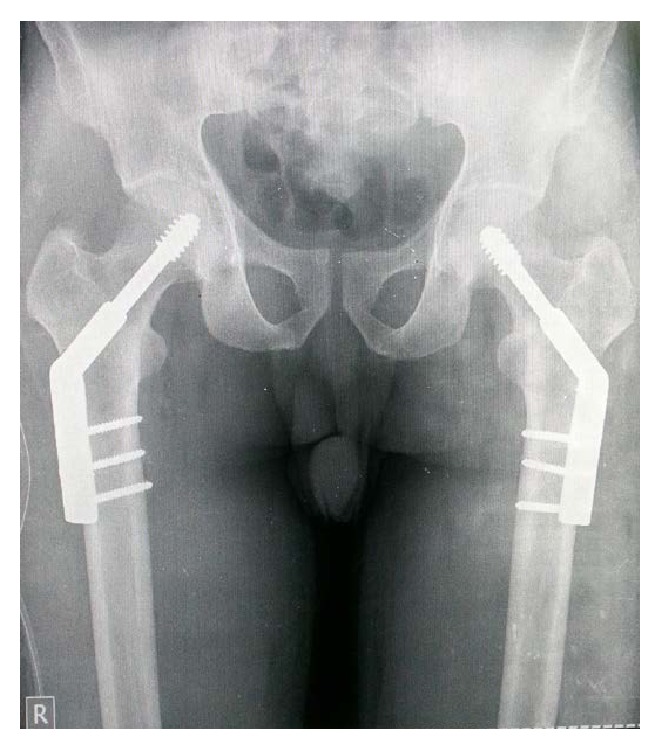
Immediate postoperative X-ray of bilateral hip joint with pelvis showing 3 holed dynamic hip screw implant in situ.

**Figure 6:**
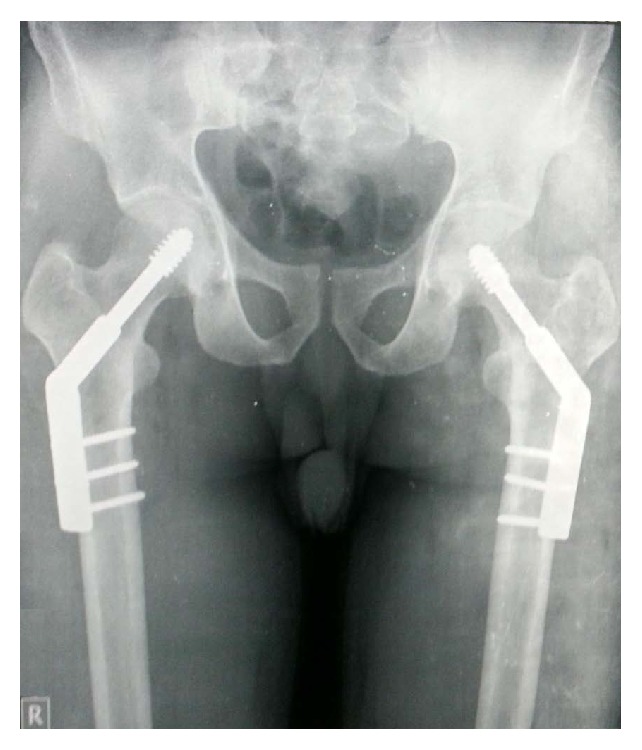
6 months postoperative X-ray of bilateral hip joint with pelvis showing 3 holed dynamic hip screw implant in situ with no articular changes.

The patient underwent surgery with internal fixation done using a 3 holed dynamic hip screw bilaterally on two sittings 2 days apart (Figure 5). Weight bearing was initiated one and half months following the surgery with the aid of a walker. At 6 months and one year postoperatively patient was doing all his activities of daily living. On radiographic examination at 6 months and one year the X-ray did not show any evidence of avascular necrosis of the femur head (Figure 6).

## 3. Discussion

Stress fracture of the femur neck was first reported by Blecher in 1905 [1] and it represents about 8% of stress fractures [2]. Stress fractures result from repetitive microtrauma; they often are classified as insufficiency and fatigue fractures. Stress placed on an abnormal bone causes insufficiency fractures to occur whereas abnormal amount of stress on normal or abnormal bone results in fatigue fracture [3]. Unilateral fractures of the femur neck have been frequently reported in literature [4–7], but few cases of bilateral fractures of the femur neck in a normal individual have been reported [8–10].

These fractures present in two distinct populations. In younger age group they are commonly seen in athletes, military recruits, and recreational runners and in the elderly individuals they occur due to osteoporosis termed as insufficiency fractures [11, 12]. Typical complaints include groin, thigh, or knee pain and pain with weight bearing relieved by non-weight bearing and can pose a diagnostic difficulty due to vague presentation of symptoms [3]. These patients are then often treated for muscle or tendon strains or early onset arthrosis of the hip joint [11]. The clinical features are also nonspecific and rotations of the hip joint are painful with internal rotation being prominently restricted and painful in these cases.

Plain radiographs sometimes fail to detect these fractures and for this reason Orcel has stated that plain radiographs are mostly useless to diagnose this condition early [10]. Magnetic resonance imaging (MRI) scan and bone scintigraphy are very sensitive in diagnosing these fractures [11, 13, 14] with magnetic resonance imaging proving to be superior to radionuclide bone scanning in providing an early and accurate diagnosis in respective studies [15, 16]. These stress fractures are of two types seen on radiography, tensile and compressive. Greater risk is there with occurrence of tensile fractures as these fractures go for displacement if undiagnosed and may later cause osteonecrosis of the femur head [17]. These fractures are thus managed surgically as they frequently progress to complete fractures of the femur neck and may become displaced [10]. Fullerton Jr. and Snowdy in their article presented a large series of femoral neck stress fractures. They present the concept that tension side fractures should be internally fixed while compression side fractures can be treated conservatively [18]. Vertical fracture patterns are more amenable to displacement due to dominant shear forces resulting in implant failure and nonunion [19, 20] which was demonstrated by Baitner et al. who compared multiple screws to the SHS for treatment of vertical-type femoral neck fracture. They found that the dynamic hip screw had less inferior femoral head displacement, less shearing displacement, and a greater load to failure when compared to the three cannulated cancellous screws [21].

To the extent of our knowledge very few cases of bilateral femoral neck fractures occurring simultaneously in a young adult with no known medical conditions have been previously reported. In our case probable reason for bilateral stress fracture could be due to lifting heavy weights on a daily basis as other causes for the stress fracture were not present; also patient was evaluated thoroughly to rule out metabolic disorders. We were also able to diagnose the fracture due to MRI done at the first assessment and thus avoid complications. The left fracture neck was complete and had to fix to prevent displacement and the fracture on the right side was fixed to prevent the fracture line from becoming complete and displaced on mobilisation as entire weight would fall on this limb. Thus we conclude that stress fracture of femur neck poses diagnostic challenge and we need high index of suspicion to diagnose this condition to prevent untoward complications.
